# Analysis of Association between the Consumer Food Quality Perception and Acceptance of Enhanced Meat Products and Novel Packaging in a Population-Based Sample of Polish Consumers

**DOI:** 10.3390/foods9111526

**Published:** 2020-10-23

**Authors:** Dominika Guzek, Dominika Głąbska, Marta Sajdakowska, Krystyna Gutkowska

**Affiliations:** 1 Department of Food Market and Consumer Research, Institute of Human Nutrition Sciences, Warsaw University of Life Sciences (WULS-SGGW), 159C Nowoursynowska Street, 02-776 Warsaw, Poland; marta_sajdakowska@sggw.edu.pl (M.S.); krystyna_gutkowska@sggw.edu.pl (K.G.); 2 Department of Dietetics, Institute of Human Nutrition Sciences, Warsaw University of Life Sciences (WULS-SGGW), 159C Nowoursynowska Street, 02-776 Warsaw, Poland; dominika_glabska@sggw.edu.pl

**Keywords:** consumer, preference, food acceptability, knowledge management, quality

## Abstract

The consumer acceptance of novel enhanced-quality products and their willingness to buy such products may be a crucial topic in the field of marketing. The aim of this study was to analyze the association between consumers’ perceptions of food quality and their acceptance of enhanced meat products and novel packaging. The study was conducted using the Computer-Assisted Personal Interview (CAPI) method in a random group of 1009 respondents, who were recruited as a representative sample based on data from the Polish National Identification Number database. The participants were asked about the most important quality determinants of food products of animal origin and about quality improvement methods and their acceptance of those methods. The quality determinants of animal-based food products were indicated as follows: origin, production technology, manufacturer, components and nutritional value, visual and sensory characteristics, expiry date, and cost. The quality improvement methods were clustered into groups that were associated with product enhancement and application of novel packaging, and the acceptance of those methods was also verified. Indicating specific quality determinants of animal-derived food products affects the consumer acceptance of product enhancement (*p* = 0.0264) and novel packaging as quality improvement methods (*p* = 0.0314). The understanding that enhancement is applied for the purpose of quality improvement did not influence the acceptance of products (*p* = 0.1582), whereas the knowledge that novel packaging is applied influenced the acceptance (*p* = 0.0044). The obtained results suggested that in the case of application of novel packaging, a higher level of knowledge may be a reason for consumer’s rejection of the resulting products, but the appearance and taste of products may contribute to the higher acceptance of novel packaging. Educating consumers may improve their acceptance of product enhancement, as concerns about the addition of food preservatives may lead them to reject enhanced products.

## 1. Introduction

Meat, as well as meat products, is an important component of a typical western diet and is consumed almost every day by some people. Although they provide essential amino acids, vitamins (mainly B_12_), and minerals (mainly iron and zinc) needed for the body, meat products, especially red and processed ones, may increase the risk of chronic diseases typical in developed countries (e.g., cardiovascular diseases, diabetes, and some cancers), as well as general mortality, when consumed in excessive amounts [1].

The role of meat and meat products in the consumer lifestyle is influenced by various factors, such as socioeconomic determinants, traditions, perceived ethics, and religious beliefs [2]. Many studies have discussed consumers’ attitudes toward meat and meat products [3,4], with increasing attention paid to the unsustainable consumption of meat and meat-derived saturated fats [5].

Nevertheless, the necessity of a transition toward more sustainable meat consumption is emphasized by nutritionists and dietitians for promoting health-related values [6]. Provision of moderate amounts of protein, from moderate proportions of meat, eggs, and dairy products, is indicated as one of the characteristics of a well-balanced diet [7]. Taking into account that Western diets often include food products of animal origin [8], reducing the intake of red and processed meat can be beneficial to both health and the environment [9].

On the one hand, plant-based products are considered as substitutes for meat, but on the other hand, less-but-better meat products are more suitable for consumers [10], as vegetarian diets may not be preferred by a majority of the population [11] and may be treated as too radical [5]. Therefore, adding more health-promoting components to meat-based food products and improving their nutritional value can be an effective strategy for reducing the intake of other meat products. This is not only due to the fact that health benefits are given more importance by consumers than environmental concerns [12] but also because a sustainable and moderate consumption of meat products is necessary.

Nowadays, consumers choose food products that satisfy their hunger as well as prevent diet-related diseases. The food market, especially the meat market, is currently undergoing many changes, and as a result, new meat products with health-promoting components appear, which are acceptable [13]. Functional foods, including meat products, providing additional health benefits [14] have been studied by many researchers, but consumer attitudes and behaviors toward such products remain a question [15,16,17].

There are three main types of factors affecting consumer behaviors—individual factors (psychological aspects), product-specific factors, and marketing factors [2]. Among the product-specific sensory factors, the most important ones are quality and sensory features, such as visual appearance, texture, flavor, and taste [18].

To obtain novel functional meat and meat products that have health-promoting properties, one of the two main approaches are used: (1) enhancing or (2) reducing the share of some components. The health-promoting components incorporated into food products of animal origin may be added either to animal fodder or directly to the product during the production process, while reducing the share of other components. However, consumers have a common assumption that all the additives added to the products are “bad” [19]. This belief is prevalent in spite of the fact that labeling food products with information about the additive, including its name and function, is mandatory, as well as that only EU-approved additives may be used in food products according to legal regulations [20]. Although labeling of food products does not change their sensory features, it could be valuable in terms of influencing consumers’ opinion about the health-promoting attributes of such products [21].

Therefore, the so-called “feed-to-food” approach (adding bioactive compounds to fodder, rather than to the product) seems to be a promising direction for improving the quality of animal-based food products to increase consumer acceptability [22,23]. However, adding bioactive components directly to products may be easier for producers. Taking this into account, an important task is to keep the initial features of a product intact [24] and sustain them during storage [25].

For maintaining the nutritional value and sensory features of animal-based food products, some advances are made in the form of novel packaging systems, which are divided into two main categories—active and intelligent packaging [26]. Although these types of packaging have existed on the market for quite some time, some consumers do not know and are still worried that innovative packaging might mislead them to buy spoiled food [27]. However, packaging may influence the consumers’ color perception of meat. As color is a major determinant of meat quality [28], various packaging systems may allow the meeting of the real market demands, providing products that may be accepted by consumers.

The present study aimed to analyze the association between consumers’ perception of food quality and their acceptance of enhanced meat products and novel packaging.

## 2. Materials and Methods

### 2.1. Study Participants

The study was conducted using the Computer-Assisted Personal Interview (CAPI) method. A total of 1009 adult Polish inhabitants were randomly recruited (inclusion criteria: age ≥ 18 years; willing to provide informed consent to participate in the study; involved in food purchasing in the household—either own or cooperative purchasing), based on data from the National Identification Number database, which is the universal electronic system for population registration. The included participants were verified to be representatives of the general population of Poland. The characteristics of the study participants are provided in Table 1.

In order to verify the representativeness of the studied sample, the distribution was compared with the characteristics of the general Polish population, as described by Statistics Poland [29]. It was verified if the proportions of gender groups were adequate when compared with the general population of adults and the studied population was stated to be representative for gender (*p* = 0.3684, Chi Square Test). Afterwards, it was verified if the proportions of age groups were adequate when compared with the general population of adults and for this assessment, it was assumed that for the youngest and oldest age group (19–29 years and ≥70 years), the proportion should be reduced, as in previous studies [16], to obtain an adequate number of respondents participating in grocery shopping. The general population of adults with indicated age groups reduced to 50% was compared with the studied population and it was stated to be representative for age (*p* = 0.5222, Chi Square Test).

### 2.2. Assessment of Consumers’ Acceptance of Enhanced Animal-Derived Food Products and Novel Packaging

The questionnaire used in the study was developed for assessing the consumers’ perception of the quality determinants of animal-based food products, quality improvement methods, and acceptance of those methods. It included questions related to the discussed issue, as presented in a previous study [17] (Table 2).

For the question on the quality determinants of food products of animal origin (Question 1), the researchers interpreted the obtained responses by clustering them in common groups associated with the same area as follows: origin (indicated by respondents as: country of origin, farming, organic farm, etc.), production technology (indicated as: certified production, process of production, technology, etc.), manufacturer (indicated as: known company, trademark, producer, etc.), components and nutritional value (indicated as: vitamins, minerals, food additives, etc.), visual and sensory characteristics (indicated as: appearance, flavor, taste, etc.), expiry date (indicated as: shelf life, fresh product, production data, etc.), and cost (indicated as: price). An additional cluster contained the answers of respondents who declared that they were not able to define the quality determinants of food products of animal origin.

Similarly, for the question about the quality improvement methods applied for the food products of animal origin (Question 3), the researchers interpreted the obtained responses by clustering them in common groups associated with the same area, which allowed analyzing of the quality improvement methods associated with product enhancement and application of novel packaging. In the cluster of methods associated with product enhancement were those responses associated with descriptions such as additives, technology of production, and so on. On the other hand, in the cluster of methods associated with the application of novel packaging were those responses associated with descriptions such as dedicated packaging, proper packaging, or specific packaging described. An additional cluster contained the respondents’ answers that were not related to the methods applied by producers (e.g., storage, not consuming products that are past the expiration date).

The structure of the interview and the order of questions allowed extending of the analysis from the most general issue associated with concept definition (what does quality mean for respondents), to market situation (what actions do they observe that can improve the quality), and finally, to the subjectively perceived acceptance (do they accept the observed actions). Thus, the data obtained from the interview were analyzed to verify the following potential associations:

- The association between the perceived quality determinants of food products of animal origin and the level of acceptance of the quality improvement methods (hypothesis: perceived quality determinants can influence the acceptance of the quality improvement methods);
- The association between the known methods of quality improvement applied for the products of animal origin and the level of acceptance of these methods (hypothesis: the known quality improvement methods are more accepted).

### 2.3. Statistical Analysis

The frequency of specific answers provided in the subgroups was compared using the Chi Square Test. The accepted level of significance was set at *p* ≤ 0.05. Statistical analysis was conducted using Statgraphics Plus for Windows 4.0 (Statistical Graphics Corporation, Rockville, MD, USA).

## 3. Results

### 3.1. Acceptance of Enhancement of Animal-Derived Food Products as a Quality Improvement Method

The acceptance of animal-derived food products’ enhancement as the quality improvement method for respondents declaring diverse quality determinants is presented in Table 3. The additional analysis conducted for the subgroups stratified by income, place of living, and educational background is presented in the Supplementary Materials (Supplementary Tables S1–S6).

It was stated that declaring diverse quality determinants influenced acceptance of the enhancement of animal-derived food products as the quality improvement method (*p* = 0.0264). In the studied group, the highest number of respondents accepted food products’ enhancement in the sub-group of respondents not able to define quality determinants (42.9%), while a lower number of respondents accepted it in the sub-groups of respondents declaring as quality determinants: origin (33.6%), production technology (26.6%), manufacturer (35.4%), components and nutritional value (32.8%), visual and sensory characteristics (26.1%), expiry date (29.7%), and cost (35.0%). At the same time, the highest number of respondents did not accept food products’ enhancement in the sub-group of respondents declaring expiry date as a quality determinant (41.5%), while a lower number of respondents did not accept it in the sub-groups of respondents declaring as quality determinants: origin (37.7%), production technology (34.4%), manufacturer (23.1%), components and nutritional value (31.2%), visual and sensory characteristics (37.0%), and cost (32.3%), or in the sub-group of respondents not being able to define quality determinants (19.8%).

The acceptance of animal-derived food products’ enhancement as a quality improvement method, for respondents declaring diverse known methods of quality improvement, is presented in Table 4.

It was stated that indicating product enhancement as a method of quality improvement did not influence acceptance of animal-derived food products’ enhancement as the quality improvement method (*p* = 0.0783). The acceptance was comparable in sub-groups of respondents indicating product enhancement, methods other than product enhancement, and those who did not know any method to improve quality.

### 3.2. Acceptance of the Application of Novel Packaging for Animal-Derived Food Products as the Quality Improvement Method

The acceptance of the application of novel packaging for animal-derived food products as the quality improvement method, for respondents declaring diverse quality determinants, is presented in Table 5. The additional analysis conducted for the sub-groups stratified by income, place of living, and educational background is presented in the Supplementary Materials (Supplementary Tables S7–S12).

It was stated that declaring diverse quality determinants influenced the acceptance of the application of novel packaging for animal-derived food products as the quality improvement method (*p* = 0.0314). In the studied group, the highest number of respondents accepted the application of novel packaging in the sub-group of respondents not able to define quality determinants (69.2%) and declaring manufacturer as a quality determinant (69.2%), while a lower number of respondents accepted it in the sub-groups of respondents declaring as quality determinants: origin (67.8%), production technology (60.9%), components and nutritional value (64.0%), visual and sensory characteristics (67.4%), expiry date (62.6%), and cost (65.0%). At the same time, the lowest number of respondents did not accept the application of novel packaging in the sub-group of respondents declaring visual and sensory characteristics (4.3%), while a higher number of respondents did not accept it in the sub-groups of respondents declaring as quality determinants: origin (16.4%), production technology (7.8%), manufacturer (9.2%), components and nutritional value (8.8%), expiry date (18.3%) and cost (18.1%), or in the sub-group of respondents not able to define quality determinants (7.7%).

The acceptance of the application of novel packaging for animal-derived food products as the quality improvement method, for respondents declaring diverse known methods of quality improvement, is presented in Table 6.

It was stated that indicating the application of novel packaging as a method of quality improvement influenced acceptance of novel packaging application for animal-derived food products as the quality improvement method (*p* = 0.0044). The acceptance was comparable in sub-groups of respondents indicating product enhancement, methods other than product enhancement, and those who did not know any method to improve quality. In the case of respondents indicating the application of novel packaging as a known method of quality improvement, the lowest number of respondents accepted the application of novel packaging (33.3%) than for the sub-group indicating methods other than novel packaging application (61.9%) and those who did not know any method to improve quality (66.1%).

## 4. Discussion

The main observation from the obtained results indicated that those individuals who do not accept the analyzed methods of product enhancement may not accept such methods probably because they equate them with the addition of food preservatives (respondents who perceived expiration date as a major determinant of quality). Meat products are traditional foods in many countries, so in order to implement dietary behavior changes in those populations, culinary knowledge and understanding of the needed changes are required, as well as providing time to adapt and accept those changes [30]. Moreover, it should be recognized that specific factors determine the perceived quality, such as the cut used and method of thermal treatment applied [31].

The low level of acceptance in this group may be a serious problem for producers and distributors. However, in the study conducted by Bearth et al. [32] in a large sample of Swiss-German households, analyzing consumers’ perception of artificial food additives, the factors that influenced the perceived level of risks and benefits were identified. The study concluded that acceptance of food additives is associated with perceived risks and benefits, which are influenced by the knowledge of legal regulations, trust in legal regulators, and a general preference for natural products [32]. Educating consumers may not only increase their knowledge of regulations but also improve their perception of food additives and minimize their worries, as education is indicated as one of the elements of intervention used for treating adult food neophobia [33].

The knowledge that product enhancement is applied does not relieve the stress resulting from the concerns of artificial additives. It is indicated that the names of food additives are sometimes even difficult to pronounce for consumers, and thus, could create an impression of unfamiliarity [22]. In spite of the fact that consumers can be provided reasonable information about risk assessment applied for food additives, it is difficult in practice [34]. Moreover, it should be emphasized that designing functional food products dedicated to specific groups of consumers with more concerns about product enhancement may be a promising strategy, but these products must be accompanied by information about health issues. Consumers with more modern health concerns (associated with the risk of diet-related diseases) tend to accept functional food products that are developed either to reduce the risk of the disease or to help in its cure [35].

The most important observation of the study was that respondents who perceived appearance and taste as major determinants of food quality accepted novel packaging methods. It must be indicated that for some animal-derived food products, appearance may be the main determinant, and therefore, applying suitable packaging is crucial. The study by Grebitus et al. [36] showed that in the case of packaged meat, consumers had a higher preference for meat having a brighter red color (aerobic packaging or carbon monoxide atmosphere packaging applied) and were willing to pay more if such a color was warranted. Nevertheless, it must be stated that consumers have higher preferences for products that they know and like [37]; therefore, the color of products must be characteristic, not changed by packaging.

The study by Giles et al. [38] indicated that improvement in taste, which was perceived as a beneficial modification of food products, especially when accompanied by a lower price, may be accepted as a reason for applying nanotechnology in food production. However, it was emphasized that nanotechnological components were accepted if they were applied only for food packaging, but not when they were integrated into food products [38].

The obtained results are in agreement with other studies conducted on carbon monoxide atmosphere packaging, which is promising for meat production [39]. In the study by Grebitus et al. [34], it was stated that a higher level of knowledge, gained through sources such as media, led to the lower acceptance of carbon monoxide atmosphere packaging.

The study by Chen et al. [40] measured the resistance to the use of new technology in food production, with an example of vacuum packaging used for beef in Canada, which is not uncommon in Europe. The information that vacuum packaging was applied influenced consumers’ choices and increased their acceptability. The authors also highlighted that, in general, there was a problem with a low share of respondents being well-informed about this technology. This suggests that, in some cases, consumers’ knowledge about the risks and benefits of the packaging system could dispel their doubts and have positive effects on market demands.

In addition, the microbiological safety of meat, which is another aspect associated with the risks and benefits of packaging, must be considered in the context of consumers’ preferences and acceptances. The perspective of consumers should be regarded irrespective of the fact that novel packaging methods could prevent quality deterioration of enhanced products, as well as reduce product spoilage, as a part of sustainable development. The study of Dastile et al. [41] emphasized that visual assessment, rather than knowledge, may be a crucial determinant of sustainable meat consumption.

In the study of Akehurst et al. [42], the gap between purchase intentions and behaviors was stated to be less visible among respondents who had a higher level of consciousness. This may be due to the fact that respondents generally underestimated the impact of the meat industry and meat consumption on the environment [43]; therefore, proper knowledge could help in transmitting attitudes into purchase decisions.

Although the study presents some novel observations in a population-based sample, it has certain limitations that must be addressed. It should be mentioned that the study analyzed only declarative acceptance of the quality improvement methods applied for food products of animal origin, and such assessment is associated with self-reporting bias [44]. This is because conscious and unconscious behaviors differ, and even if the respondents do not intend to report false answers, their responses may be different from their unconscious preferences [45].

## 5. Conclusions

The results obtained in the study indicated that for the application of novel packaging, a higher level of knowledge may be a reason for consumers’ rejection of the resulting products, but the appearance and taste of products may contribute to the higher acceptance of novel packaging. Educating consumers may improve their acceptance of product enhancement, as concerns about the addition of food preservatives, due to insufficient knowledge, may lead them to reject enhanced products.

## Figures and Tables

**Table 1:** The characteristics of the included participants of the study (*n* = 1009).

Characteristic		N	%
Gender	men	496	49.2
	women	513	50.8
Age (years)	19–29	121	12.0
	30–39	220	21.8
	40–49	211	20.9
	50–59	204	20.2
	60–70	181	17.9
	≥70	72	7.1
Educational background *	primary	120	12.1
	vocational	342	34.5
	secondary	377	38.0
	higher	152	15.3
* Per capita * net income *	<1500 PLN (<350 EUR)	108	18.5
	1500–2500 PLN (~350–600 EUR)	153	26.2
	2500–4000 PLN (~600–1000 EUR)	205	35.0
	>4000 PLN (>1000 EUR)	119	20.3
Village/city (number of inhabitants) *	village	352	35.1
	city < 20,000	133	13.3
	city 20,000–100,000	203	20.3
	city 100,000–500,000	201	20.1
	city > 500,000	113	11.3
Household size (number of persons) *	1	123	13.0
	2	265	27.9
	3	241	25.4
	4	209	22.0
	≥5	111	11.7

* Missing data in category: educational background *n* = 18; per capita net income *n* = 424; town of residence *n* = 7; household size *n* = 60; PLN—Polish Zloty (Polish currency).

**Table 2:** The questionnaire applied in the study.

Question That Was Asked	Characteristics of Question	Interpretation of Answers
(1) Please define the quality determinant of food products of animal origin which is in your opinion the most important one	Open-ended question	If respondent defined more than one determinant, the first one listed was interpreted as the most important.
(2) Do you know any quality improvement methods applied for food products of animal origin?	Yes/no question	If respondent stated that he knows any quality improvement method, he was asked to describe the method which he knows.
(3) Please describe the quality improvement method applied for food products of animal origin which you know.	Open-ended question	Respondents were allowed to describe an unlimited number of methods, if they wanted to.
(4) What is your level of acceptance of the quality improvement/assurance methods applied for food products of animal origin, for the following groups of actions? - Actions associated with origin (e.g., method of farming); - Actions associated with production technology (e.g., certification of production); - Actions associated with manufacturer (e.g., trademark); - Actions associated with components and nutritional value (e.g., food additives); - Actions associated with visual and sensory characteristics (e.g., flavor); - Actions associated with expiry date (e.g., shelf life); - Actions associated with cost of production (e.g., price).	Close-ended questions (7-point scale): 1: Definitely do not accept; 2: Moderately do not accept; 3: Slightly do not accept; 4: Undecided; 5: Slightly accept; 6: Moderately accept; 7: Definitely accept.	1–3: Negative answers; 4: Neutral answer; 5–7: Positive answers.

**Table 3:** Level of acceptance of enhanced animal-derived food products for respondents stratified by perceived quality determinants.

Perceived Quality Determinants	Acceptance of Animal-Derived Food Products’ Enhancement as the Quality Improvement Method								*p*
	1 *	2	3	4	5	6	7	0	
Origin	10 (15.6%)	3 (4.7%)	4 (6.3%)	13 (20.3%)	11 (17.2%)	7 (10.9%)	10 (15.6%)	6 (9.4%)	0.0264
Production technology	25 (17.2%)	12 (8.2%)	12 (8.2%)	24 (16.4%)	30 (20.5%)	10 (6.8%)	25 (17.2%)	8 (5.5%)	
Manufacturer	9 (19.6%)	2 (4.3%)	3 (6.5%)	11 (23.9%)	7 (15.2%)	2 (4.3%)	7 (15.3%)	5 (10.9%)	
Components and nutritional value	32 (14.2%)	17 (7.5%)	14 (6.2%)	49 (21.6%)	41 (18.1%)	32 (14.2%)	25 (11.1%)	16 (7.1%)	
Visual and sensory characteristics	20 (16.0%)	9 (7.2%)	9 (7.2%)	18 (14.4%)	23 (18.4%)	15 (12.0%)	19 (15.2%)	12 (9.6%)	
Expiry date	52 (21.1%)	14 (5.7%)	18 (7.3%)	46 (18.7%)	32 (13.1%)	16 (6.5%)	37 (15.0%)	31 (12.6%)	
Cost	7 (10.8%)	0 (0.0%)	2 (3.1%)	21 (32.3%)	13 (20.0%)	9 (13.8%)	6 (9.2%)	7 (10.8%)	
Not able to define quality determinants	9 (9.9%)	2 (2.2%)	5 (5.5%)	22 (24.2%)	13 (14.2%)	10 (11.0%)	16 (17.6%)	14 (15.4%)	

* 1–3 negative answers (1—definitely; 2—moderately; 3—slightly do not accept); 4—neutral answer; 5–7 positive answers (5—slightly; 6—moderately; 7—definitely accept); 0—do not observe such method for food products of animal origin in Poland.

**Table 4:** Level of acceptance of enhanced animal-derived food products for respondents stratified by known methods of quality improvement.

Known Methods of Quality Improvement	Acceptance of Animal-Derived Food Products’ Enhancement as the Quality Improvement Method								*p*
	1 *	2	3	4	5	6	7	0	
Indicates product enhancement	122 (14.8%)	45 (5.5%)	58 (7.0%)	170 (20.6%)	145 (17.6%)	76 (9.2%)	119 (14.4%)	90 (10.9%)	0.0783
Indicates method other than product enhancement	37 (25.2%)	13 (8.8%)	7 (4.8%)	26 (17.7%)	20 (13.6%)	17 (11.6%)	20 (13.6%)	7 (4.8%)	
Does not know any method to improve quality	5 (13.5%)	1 (2.7%)	2 (5.4%)	8 (21.6%)	5 (13.5%)	8 (21.6%)	6 (16.2%)	2 (5.4%)	

* 1–3 negative answers (1—definitely; 2—moderately; 3—slightly do not accept); 4—neutral answer; 5–7 positive answers (5—slightly; 6—moderately; 7—definitely accept); 0—do not observe such method for food products of animal origin in Poland.

**Table 5:** Level of acceptance of novel packaging in animal-derived food products for respondents stratified by perceived quality determinants.

Perceived Quality Determinants	Acceptance of the Application of Novel Packaging for Animal-Derived Food Products as the Quality Improvement Method								*p*
	1 *	2	3	4	5	6	7	0	
Origin	1 (1.6%)	2 (3.1%)	2 (3.1%)	18 (28.1%)	10 (15.6%)	7 (10.9%)	22 (34.5%)	2 (3.1%)	
Production technology	10 (6.8%)	6 (4.1%)	8 (5.5%)	20 (13.7%)	35 (24.0%)	20 (13.7%)	44 (30.1%)	3 (2.1%)	
Manufacturer	2 (4.3%)	0 (0.0%)	0 (0.0%)	10 (21.7%)	9 (19.6%)	2 (4.3%)	20 (43.6%)	3 (6.5%)	
Components and nutritional value	23 (10.2%)	6 (2.7%)	12 (5.3%)	34 (15.0%)	45 (19.9%)	42 (18.6%)	60 (26.5%)	4 (1.8%)	0.0314
Visual and sensory characteristics	4 (3.2%)	0 (0.0%)	7 (5.6%)	31 (24.8%)	21 (16.8%)	18 (14.4%)	41 (32.8%)	3 (2.4%)	
Expiry date	29 (11.8%)	6 (2.4%)	10 (4.1%)	40 (16.3%)	44 (17.9%)	38 (15.4%)	72 (29.3%)	7 (2.8%)	
Cost	3 (4.6%)	1 (1.5%)	2 (3.1%)	9 (13.8%)	17 (26.2%)	13 (20.0%)	15 (23.1%)	5 (7.7%)	
Does not know what determines food quality	4 (4.4%)	2 (2.2%)	1 (1.1%)	16 (17.6%)	21 (23.1%)	18 (19.8%)	24 (26.3%)	5 (5.5%)	

* 1–3 negative answers (1—definitely; 2—moderately; 3—slightly do not accept); 4—neutral answer; 5–7 positive answers (5—slightly; 6—moderately; 7—definitely accept); 0—do not observe such method for food products of animal origin in Poland.

**Table 6:** Level of acceptance of novel packaging for animal-derived food products for respondents stratified by known methods of quality improvement.

Known Methods of Quality Improvement	Acceptance of the Application of Novel Packaging for Animal-Derived Food Products as the Quality Improvement Method								*p*
	1 *	2	3	4	5	6	7	8	
Indicates application of novel packaging	58 (7.0%)	20 (2.4%)	35 (4.2%)	140 (17.0%)	176 (21.3%)	132 (16.0%)	237 (28.7%)	27 (3.3%)	0.0044
Indicates other method than the application of novel packaging	18 (9.9%)	2 (1.1%)	6 (3.3%)	38 (21.0%)	26 (14.4%)	25 (13.8%)	61 (33.7%)	5 (2.8%)	
Does not know any method to improve quality	0 (0.0%)	1 (33.3%)	1 (33.3%)	0 (0.0%)	0 (0.0%)	1 (33.3%)	0 (0.0%)	0 (0.0%)	

* 1–3 negative answers (1—definitely; 2—moderately; 3—slightly do not accept); 4—neutral answer; 5–7 positive answers (5—slightly; 6—moderately; 7—definitely accept); 0—do not observe such method for food products of animal origin in Poland.

## Supplementary Materials

The following are available online at https://www.mdpi.com/2304-8158/9/11/1526/s1. Supplementary Table S1. Acceptance of the enhancement of animal-derived food products as the quality improvement method, for low income respondents who declared diverse quality determinants (*n* = 261). Supplementary Table S2. Acceptance of the enhancement of animal-derived food products as the quality improvement method, for high income respondents who declared diverse quality determinants (*n* = 324). Supplementary Table S3. Acceptance of the enhancement of animal-derived food products as the quality improvement method, for respondents of primary and vocational education level who declared diverse quality determinants (*n* = 462). Supplementary Table S4. Acceptance of the enhancement of animal-derived food products as the quality improvement method, for respondents of secondary and higher education level respondents who declared diverse quality determinants (*n* = 529). Supplementary Table S5. Acceptance of the enhancement of animal-derived food products as the quality improvement method, for respondents living in cities and villages of less than 100,000 inhabitants who declared diverse quality determinants (*n* = 688). Supplementary Table S6. Acceptance of the enhancement of animal-derived food products as the quality improvement method, for respondents living in cites of more than 100,000 inhabitants who declared diverse quality determinants (*n* = 314). Supplementary Table S7. Acceptance of the application of novel packaging for animal-derived food products as the quality improvement method, for low income respondents who declared diverse quality determinants (*n* = 261). Supplementary Table S8. Acceptance of the application of novel packaging for animal-derived food products as the quality improvement method, for high income respondents who declared diverse quality determinants (*n* = 324). Supplementary Table S9. Acceptance of the application of novel packaging for animal-derived food products as the quality improvement method, for respondents of primary and vocational education level declaring diverse quality determinants (*n* = 462). Supplementary Table S10. Acceptance of the application of novel packaging for animal-derived food products as the quality improvement method, for respondents of secondary and higher education level who declared diverse quality determinants (*n* = 529). Supplementary Table S11. Acceptance of the application of novel packaging for animal-derived food products as the quality improvement method, for respondents living in cities and villages of less than 100,000 inhabitants who declared diverse quality determinants (*n* = 688). Supplementary Table S12. Acceptance of the application of novel packaging for animal-derived food products as the quality improvement method, for respondents living in cities of more than 100,000 inhabitants who declared diverse quality determinants (*n* = 314).
