# Supplementary material for: Analysis of Association between the Consumer Food Quality Perception and Acceptance of Enhanced Meat Products and Novel Packaging in a Population-Based Sample of Polish Consumers
Source: Foods. 2020 Oct 23;9(11):1526. doi: 10.3390/foods9111526 (PMC7690865; doi:10.3390/foods9111526)
Supplement: Supplementary file 1 [file foods-09-01526-s001.pdf]

foods

Supplementary Materials

Analysis of Association Between the Consumer Food Quality Perception and Acceptance of Enhanced Meat Products and Novel Packaging in a Population-Based Sample of Polish Consumers

Dominika Guzek [1,*], Dominika Głąbska [2], Marta Sajdakowska [1] and Krystyna Gutkowska [1]

Supplementary Table 1. Acceptance of the animal-derived food products enhancing as the quality improvement method, for low income respondents who declared diverse quality determinants (n=261).

Declared quality determinants	Acceptance of the animal-derived food products enhancing as the quality improvement method				*p*
	Disapprove	Neutral	Approve	NA	
Origin	2 (18.2%)	2 (18.2%)	5 (45.5%)	2 (18.2%)	<0.0001
Production technology	6 (40.0%)	5 (33.3%)	3 (20.0%)	1 (6.7%)	
Manufacturer	27 (49.1%)	0 (0.0%)	21 (38.2%)	7 (12.7%)	
Components and nutritional value	5 (14.3%)	12 (34.3%)	14 (40.0%)	4 (11.4%)	
Visual and sensory characteristics	3 (11.1%)	18 (66.7%)	3 (11.1%)	3 (11.1%)	
Expiry date	15 (28.8%)	17 (32.7%)	16 (30.8%)	4 (7.7%)	
Cost	5 (17.9%)	9 (32.1%)	10 (35.7%)	4 (14.3%)	
Not able to define quality determinants	9 (23.7%)	8 (21.1%)	14 (36.8%)	7 (18.4%)	

*NA: do not observe such method for food products of animal origin in Poland.

Supplementary Table 2. Acceptance of the animal-derived food products enhancing as the quality improvement method, for high income respondents who declared diverse quality determinants (n=324).

Declared quality determinants	Acceptance of the animal-derived food products enhancing as the quality improvement method				*p*
	Disapprove	Neutral	Approve	NA	
Origin	8 (47.1%)	4 (23.5%)	4 (23.5%)	1 (5.9%)	0.0031
Production technology	14 (33.3%)	11 (26.2%)	15 (35.7%)	2 (4.8%)	
Manufacturer	8 (28.6%)	8 (28.6%)	3 (10.7%)	9 (32.1%)	
Components and nutritional value	29 (31.9%)	23 (25.3%)	35 (38.5%)	4 (4.4%)	
Visual and sensory characteristics	17 (31.5%)	12 (22.2%)	18 (33.3%)	7 (13.0%)	
Expiry date	27 (45.8%)	10 (16.9%)	22 (37.3%)	0 (0.0%)	
Cost	4 (25.0%)	2 (12.5%)	8 (50.0%)	2 (12.5%)	
Not able to define quality determinants	5 (29.4%)	4 (23.5%)	7 (41.2%)	1 (5.9%)	

*NA: do not observe such method for food products of animal origin in Poland.

Supplementary Table 3. Acceptance of the animal-derived food products enhancing as the quality improvement method, for respondents of primary and vocational education level who declared diverse quality determinants (n=462).

Declared quality determinants	Acceptance of the animal-derived food products enhancing as the quality improvement method				*p*
	Disapprove	Neutral	Approve	NA	
Origin	6 (31.6%)	5 (26.3%)	6 (31.6%)	2 (10.5%)	0.2013
Production technology	20 (30.8%)	13 (20.0%)	22 (33.8%)	10 (15.4%)	
Manufacturer	8 (42.1%)	2 (10.5%)	4 (21.1%)	5 (26.3%)	
Components and nutritional value	28 (29.5%)	25 (26.3%)	38 (40.0%)	4 (4.2%)	
Visual and sensory characteristics	15 (28.3%)	13 (24.5%)	19 (35.8%)	6 (11.3%)	
Expiry date	51 (37.2%)	28 (20.4%)	39 (28.5%)	19 (13.9%)	
Cost	4 (15.4%)	7 (26.9%)	11 (42.3%)	4 (15.4%)	
Not able to define quality determinants	8 (16.7%)	13 (27.1%)	22 (45.8%)	5 (10.4%)	

*NA: do not observe such method for food products of animal origin in Poland.

Supplementary Table 4. Acceptance of the animal-derived food products enhancing as the quality improvement method, for respondents of secondary and higher education level respondents who declared diverse quality determinants (n=529).

Declared quality determinants	Acceptance of the animal-derived food products enhancing as the quality improvement method				*p*
	Disapprove	Neutral	Approve	NA	
Origin	16 (36.4%)	11 (25.0%)	11 (25.0%)	6 (13.6%)	0.1568
Production technology	35 (43.2%)	12 (14.8%)	27 (33.3%)	7 (8.6%)	
Manufacturer	9 (33.3%)	9 (33.3%)	8 (29.6%)	1 (3.7%)	
Components and nutritional value	42 (33.9%)	37 (29.8%)	40 (32.3%)	5 (4.0%)	
Visual and sensory characteristics	24 (34.3%)	15 (21.4%)	21 (30.0%)	10 (14.3%)	
Expiry date	50 (46.3%)	16 (14.8%)	34 (31.5%)	8 (7.4%)	
Cost	10 (29.4%)	10 (29.4%)	11 (32.4%)	3 (8.8%)	
Not able to define quality determinants	10 (24.4%)	9 (22.0%)	16 (39.0%)	6 (14.6%)	

*NA: do not observe such method for food products of animal origin in Poland.

Supplementary Table 5. Acceptance of the animal-derived food products enhancing as the quality improvement method, for respondents living in cities and villages of less than 100,000 inhabitants who declared diverse quality determinants (n=688).

Declared quality determinants	Acceptance of the animal-derived food products enhancing as the quality improvement method				*p*
	Disapprove	Neutral	Approve	NA	
Origin	7 (35.0%)	5 (25.0%)	3 (15.0%)	5 (25.0%)	0.3104
Production technology	15 (38.5%)	7 (17.9%)	12 (30.8%)	5 (12.8%)	
Manufacturer	8 (28.6%)	7 (25%)	10 (35.7%)	3 (10.7%)	
Components and nutritional value	22 (29.7%)	19 (25.7%)	29 (39.2%)	4 (5.4%)	
Visual and sensory characteristics	10 (28.6%)	8 (22.9%)	13 (37.1%)	4 (11.4%)	
Expiry date	25 (38.5%)	10 (15.4%)	21 (32.3%)	9 (13.8%)	
Cost	4 (14.3%)	11 (39.3%)	10 (35.7%)	3 (10.7%)	
Not able to define quality determinants	3 (12.0%)	7 (28.0%)	12 (48.0%)	3 (12.0%)	

*NA: do not observe such method for food products of animal origin in Poland.

Supplementary Table 6. Acceptance of the animal-derived food products enhancing as the of the quality improvement method, for respondents living in cites of more than 100,000 inhabitants who declared diverse quality determinants (n=314).

Declared quality determinants	Acceptance of the animal-derived food products enhancing as the of the quality improvement method				*p*
	Disapprove	Neutral	Approve	NA	
Origin	15 (34.1%)	11 (25.0%)	14 (31.8%)	4 (9.1%)	0.2359
Production technology	40 (37.4%)	18 (16.8%)	37 (34.6%)	12 (11.2%)	
Manufacturer	9 (50.0%)	4 (22.2%)	2 (11.1%)	3 (16.7%)	
Components and nutritional value	29 (32.2%)	20 (22.2%)	28 (31.1%)	13 (14.4%)	
Visual and sensory characteristics	50 (34.0%)	41 (27.9%)	49 (33.3%)	7 (4.8%)	
Expiry date	77 (42.5%)	34 (18.8%)	52 (28.7%)	18 (9.9%)	
Cost	11 (29.7%)	9 (24.3%)	13 (35.1%)	4 (10.8%)	
Not able to define quality determinants	14 (21.9%)	15 (23.4%)	27 (42.2%)	8 (12.5%)	

*NA: do not observe such method for food products of animal origin in Poland.

Supplementary Table 7. Acceptance of the novel packaging applying for animal-derived food products as the quality improvement method, for low income respondents who declared diverse quality determinants (n=261).

Declared quality determinants	Acceptance of the novel packaging applying for animal-derived food products as the quality improvement method				*p*
	**Disapprove**	**Neutral**	**Approve**	**NA**	
Origin	1 (6.7%)	4 (26.7%)	10 (66.7%)	0 (0.0%)	0.1037
Production technology	6 (15.8%)	4 (10.5%)	27 (71.1%)	1 (2.6%)	
Manufacturer	1 (11.1%)	2 (22.2%)	6 (66.7%)	0 (0.0%)	
Components and nutritional value	9 (17.3%)	10 (19.2%)	33 (63.5%)	0 (0.0%)	
Visual and sensory characteristics	2 (7.1%)	3 (10.7%)	23 (82.1%)	0 (0.0%)	
Expiry date	12 (16.4%)	9 (12.3%)	51 (69.9%)	1 (1.4%)	
Cost	1 (9.1%)	1 (9.1%)	7 (63.6%)	2 (18.2%)	
Not able to define quality determinants	2 (5.7%)	6 (17.1%)	23 (65.7%)	4 (11.4%)	

*NA: do not observe such method for food products of animal origin in Poland.

Supplementary Table 8. Acceptance of the novel packaging applying for animal-derived food products as the quality improvement method, for high income respondents who declared diverse quality determinants (n=324).

Declared quality determinants	Acceptance of the novel packaging applying for animal-derived food products as the of the quality improvement method				*p*
	**Disapprove**	**Neutral**	**Approve**	**NA**	
Origin	3 (17.6%)	5 (29.4%)	8 (47.1%)	1 (5.9%)	0.0794
Production technology	5 (11.9%)	3 (7.1%)	34 (81.0%)	0 (0.0%)	
Manufacturer	1 (5.3%)	6 (31.6%)	12 (63.2%)	0 (0.0.%)	
Components and nutritional value	18 (19.8%)	9 (9.9%)	61 (67.0%)	3 (3.3%)	
Visual and sensory characteristics	3 (5.6%)	14 (25.9%)	36 (66.7%)	1 (1.9%)	
Expiry date	12 (17.6%)	8 (11.8%)	47 (69.1%)	1 (1.5%)	
Cost	2 (12.5%)	2 (12.5%)	11 (68.8%)	1 (6.3%)	
Not able to define quality determinants	0 (0.0%)	4 (23.5%)	13 (76.5%)	0 (0.0%)	

*NA: do not observe such method for food products of animal origin in Poland.

Supplementary Table 9. Acceptance of the novel packaging applying for animal-derived food products as the quality improvement method, for respondents of primary and vocational education level declaring diverse quality determinants (n=462).

Declared quality determinants	Acceptance of the novel packaging applying for animal-derived food products as the quality improvement method				*p*
	**Disapprove**	**Neutral**	**Approve**	**NA**	
Origin	0 (0.0%)	5 (26.3%)	14 (73.7%)	0 (0.0%)	0.0266
Production technology	10 (15.4%)	14 (21.5%)	39 (60.0%)	2 (3.1%)	
Manufacturer	1 (5.3%)	2 (10.5%)	13 (68.4%)	3 (15.8%)	
Components and nutritional value	17 (17.9%)	13 (13.7%)	64 (67.4%)	1 (1.1%)	
Visual and sensory characteristics	5 (9.4%)	9 (17.0%)	39 (73.6%)	0 (0.0%)	
Expiry date	18 (13.1%)	24 (17.5%)	90 (65.7%)	5 (3.6%)	
Cost	1 (3.8%)	2 (7.7%)	19 (73.1%)	4 (15.4%)	
Not able to define quality determinants	5 (10.4%)	8 (16.7%)	33 (68.8%)	2 (4.2%)	

*NA: do not observe such method for food products of animal origin in Poland.

Supplementary Table 10. Acceptance of the novel packaging applying for animal-derived food products as the quality improvement method, for respondents of secondary and higher education level who declared diverse quality determinants (n=529).

Declared quality determinants	Acceptance of the novel packaging applying for animal-derived food products as the quality improvement method				*p*
	**Disapprove**	**Neutral**	**Approve**	**NA**	
Origin	5 (11.4%)	12 (27.3%)	25 (56.8%)	2 (4.5%)	0.0050
Production technology	14 (17.3%)	6 (7.4%)	60 (74.1%)	1 (1.2%)	
Manufacturer	1 (3.7%)	8 (29.6%)	18 (66.7%)	0 (0.0%)	
Components and nutritional value	22 (17.7%)	20 (16.1%)	80 (64.5%)	2 (1.6%)	
Visual and sensory characteristics	5 (7.1%)	22 (31.4%)	41 (58.6%)	2 (2.9%)	
Expiry date	26 (24.1%)	16 (14.8%)	64 (59.3%)	2 (1.9%)	
Cost	4 (11.8%)	6 (17.6%)	23 (67.6%)	1 (2.9%)	
Not able to define quality determinants	2 (4.9%)	7 (17.1%)	29 (70.7%)	3 (7.3%)	

Supplementary Table 11. Acceptance of the novel packaging applying for animal-derived food products as the quality improvement method, for respondents living in cities and villages of less than 100,000 inhabitants who declared diverse quality determinants (n=688).

Declared quality determinants	Acceptance of the novel packaging applying for animal-derived food products as the of the quality improvement method				*p*
	Disapprove	Neutral	Approve	NA	
Origin	3 (6.8%)	11 (25.0%)	28 (63.6%)	2 (4.5%)	0.1308
Production technology	18 (16.8%)	10 (9.3%)	76 (71.0%)	3 (2.8%)	
Manufacturer	2 (11.1%)	4 (22.2%)	12 (66.7%)	0 (0.0%)	
Components and nutritional value	28 (19%)	18 (12.2%)	98 (66.7%)	3 (2.0%)	
Visual and sensory characteristics	8 (8.9%)	21 (23.3%)	60 (66.7%)	1 (1.1%)	
Expiry date	38 (21.0%)	30 (16.6%)	107 (59.1%)	6 (3.3%)	
Cost	4 (10.8%)	5 (13.5%)	26 (70.3%)	2 (5.4%)	
Not able to define quality determinants	7 (10.9%)	11 (17.2%)	42 (65.6%)	4 (6.3%)	

*NA: do not observe such method for food products of animal origin in Poland.

Supplementary Table 12. Acceptance of the novel packaging applying for animal-derived food products as the of the quality improvement method, for respondents living in cities of more than 100,000 inhabitants who declared diverse quality determinants (n=314).

Declared quality determinants	Acceptance of the novel packaging applying for animal-derived food products as the quality improvement method				*p*
	Disapprove	Neutral	Approve	NA	
Origin	2 (10.0%)	7 (35.0%)	11 (55.0%)	0 (0.0%)	0.0994
Production technology	6 (15.4%)	10 (25.6%)	23 (59.0%)	0 (0.0%)	
Manufacturer	0 (0.0%)	6 (21.4%)	19 (67.9%)	3 (10.7%)	
Components and nutritional value	12 (16.2%)	14 (18.9%)	47 (63.5%)	1 (1.4%)	
Visual and sensory characteristics	3 (8.6%)	10 (28.6%)	20 (57.1%)	2 (5.7%)	
Expiry date	7 (10.8%)	10 (15.4%)	47 (72.3%)	1 (1.5%)	
Cost	2 (7.1%)	4 (14.3%)	19 (67.9%)	3 (10.7%)	
Not able to define quality determinants	0 (0.0%)	4 (16.0%)	20 (80.0%)	1 (4.0%)	

*NA: do not observe such method for food products of animal origin in Poland.

© 2020 by the authors. Submitted for possible open access publication under the terms and conditions of the Creative Commons Attribution (CC BY) license (http://creativecommons.org/licenses/by/4.0/).
